# Iron-rich foods consumption and its associated factors among children aged 6–23 months in South and Southeast Asia: a multilevel analysis of demographic and health surveys

**DOI:** 10.1017/S1368980025101626

**Published:** 2025-12-26

**Authors:** Melaku Tadege Engidaw, Prasenjit Mondal, Patricia Lee, Qonita Rachmah, Faruk Ahmed

**Affiliations:** 1 Public Health, School of Medicine and Dentistry, Griffith University, Gold Coast Campus, Gold Coast, QLD 4222, Australia; 2 Department of Public Health (Human Nutrition), College of Health Sciences, Debre Tabor University, Debre Tabor, Ethiopia; 3 Department of Medical Research, China Medical University Hospital, Taichung City, Taiwan; 4 Department of Nutrition, Faculty of Public Health, Universitas Airlangga, Surabaya, Indonesia

**Keywords:** Iron-rich foods, Consumption, Iron deficiency anaemia, Children, South and Southeast Asia

## Abstract

**Objective:**

This study assessed iron-rich food consumption and its associated factors among children aged 6–23 months in South and Southeast Asia.

**Design:**

A cross-sectional study from the Standard Demographic and Health Survey (2015–2022).

**Setting:**

South and Southeast Asian countries.

**Participants:**

Data collected from 95 515 children aged 6–23 months, including information from their parents or caregivers.

**Results:**

The overall proportion of children, aged 6–23 months, consuming iron-rich foods in the region was 29·87 % (95 % CI: 29·58, 30·16). Higher odds of iron-rich food consumption were observed among children aged 12–23 months (adjusted OR (AOR) = 3·59; 95 % CI: 3·45, 3·76), had history of exclusive breast-feeding (AOR = 1·17; 95 % CI: 1·12, 1·23), born to teenage motherhood (AOR = 1·09; 95 % CI: 1·02, 1·17), born in health institution (AOR = 1·10; 95 % CI: 1·02, 1·19) and had pregnant mother at the time of the survey (AOR = 1·60; 95 % CI: 1·50, 1·72). Children of birth order 2–4 (AOR = 1·26; 95 % CI: 1·20, 1·32) and 5+ (AOR = 1·29; 95 % CI: 1·18, 1·43), from female-headed households (AOR = 1·06; 95 % CI: 1·01, 1·12) and those with household mass media exposure (AOR = 1·27; 95 % CI: 1·19, 1·36) also had significantly higher odds of iron-rich food consumption. Additionally, higher odds ratio (OR) (AOR > 1) of iron-rich food consumption were observed in Cambodia, Bangladesh, Indonesia, Myanmar, Maldives, Philippines, Pakistan and Timor-Leste.

**Conclusion:**

Across countries, only about 30 % of children consumed iron-rich foods, with significant variation. Targeted public health efforts are essential to address maternal, child and household factors that influence intake.

Anaemia is a significant public health problem worldwide, affecting 269 million children. In 2019, the global prevalence of anaemia among children aged 6–59 months was 39·8 %^(1)^. The severity of the problem is higher in low- and middle-income countries^(1,2)^. In 2023, Southeast Asian countries reported the highest regional prevalence of anaemia. In this region, anaemia affects 57·3 % of individuals across all age groups^(3)^. Although the aetiology of anaemia varies widely, about half of the anaemia is considered due to iron deficiency^(4)^, primarily due to insufficient consumption of iron-rich foods, especially among vulnerable groups like women and children^(5)^. Among under-5 children, children aged 6–23 months face heightened vulnerability to iron deficiency anaemia^(6)^. Iron deficiency anaemia negatively impacts the overall well-being, growth and cognitive development of children, along with increasing susceptibility to infections, affecting their school performance^(7,8)^.

According to infant and young child feeding (IYCF) guidelines, the timing for introducing complementary foods to young children is 6 months of age^(9)^. In low- and middle-income countries, children of this age group often have poor diets lacking nutritional value and diversity, with only a small proportion receiving safe and nutritionally adequate complementary foods^(10)^.

Preventive measures for anaemia or iron deficiency anaemia in children from developing countries commonly include iron supplementation and deworming^(11)^. However, despite these interventions, anaemia continues to be a significant public health problem among children worldwide, particularly in sub-Saharan Africa (SSA) and Southeast Asia^(12)^. The WHO recommends providing children with two daily servings of iron-rich complementary foods, such as fortified cereals and mashed meats^(13,14)^ to enhance their iron intake. Nevertheless, globally, more than half of children fail to consume sufficient iron-rich foods^(15)^.

A wide range of factors contribute to inadequate micronutrient intake among children. These include geographical location^(16)^, racial differences, lower level of maternal education, household food insecurity^(17)^, monotonous diets, low bioavailability, seasonal variations and the consumption of phytate-rich foods as staples^(18,19)^. Other factors include maternal awareness, child age, religion, household wealth status and feeding practices^(20,21)^. Moreover, the consumption of iron-rich foods among children is influenced by sociodemographic (maternal age and education level) and household-level factors (family size, wealth status and mass media exposure)^(22)^. Additionally, individual-level factors such as low antenatal care (ANC) visits, institutional delivery, child’s age, gender and birth order, as well as community-level variables such as regional differences and overall level of women’s literacy at the community level, showed a strong association with iron consumption among children aged 6–59 months^(21,23,24)^. To better illustrate the range of factors that directly or indirectly influence the consumption of iron-rich foods among children, a conceptual framework was modified from a previous similar study in SSA^(25)^ (see online supplementary material, Supplemental Figure 1).

Furthermore, despite the high burden of childhood anaemia in South and Southeast Asia, data on dietary iron intake among children aged 6–23 months in this region remain limited. For example, studies in Thailand have shown that 35·9 % of children had inadequate iron intake^(26)^, with 35 % to 80 % not meeting the recommended dietary requirements^(27)^. Another study in Pakistan found that children consumed an average of only 2·6 mg of iron per d, which is substantially below the recommended daily intake of 7–10 mg^(28)^. Additionally, a regional analysis identified iron as a ‘problem nutrient’ among children aged 6–23 months across five Southeast Asian countries, indicating that diets fail to meet nutritional recommendations^(29)^.

These findings highlight a significant evidence gap and emphasise the need for comprehensive, multi-country research to better understand and address inadequate iron intake and its associated factors among young children in South and Southeast Asia. Then, this study addresses the gap by examining individual and contextual factors associated with iron-rich food intake among children aged 6–23 months in South and Southeast Asia, based on the hypothesis that household characteristics and broader community influences significantly shape children’s dietary iron consumption.

## Methods

### Data sources

The recent Demographic and Health Survey (DHS) data from South and Southeast Asian countries were collected between 2015 and 2022. After a formal written request, the DHS program (https://www.dhsprogram.com/) officially granted permission for the use of data for this analysis.

The DHS program employs a multistage cluster sampling technique. The primary sampling units in DHS surveys are enumeration areas (EA), which are geographic clusters of households (HH) defined during the most recent national census. The primary sampling units are stratified by both urban and rural areas, as well as the first level of administrative or geographic regions within the country. DHS sampling is conducted in two stages. First, the primary sampling units or EA are selected with probability proportional to their population size, based on the most recent national census. At the second stage, systematic sampling of HH is done in each of the selected clusters based on an updated HH listing.

In each country, participants in each cluster were nested within regions of the nation, which is a hierarchical structure. From all fourteen South and Southeast Asian countries, the datasets of eleven countries (namely Afghanistan, Bangladesh, Cambodia, India, Indonesia, the Maldives, Myanmar, Nepal, Pakistan, the Philippines and Timor-Leste) were included in this analysis. The remaining three countries were excluded from this study due to the unavailability of recent DHS datasets (namely Sri Lanka, Thailand and Vietnam had their last data collected in 1987, 1987 and 2002, respectively)^(30)^.

### Study population

For this study, the study population consisted of all children aged 6–23 months living with their mothers or caregivers. The DHS collects data from only one eligible child (aged 6–23 months) per household. If there is more than one eligible child, the index child (the youngest) was selected for data collection.

### Study variables

The outcome variable for this study was the consumption of iron-rich foods within the past 24 h, measured as either ‘yes’ or ‘no’. A child aged 6–23 months was classified as having iron-rich food consumption if they ate at least one item from a list of four (eggs, meat, organ meat or fish) during that period. If none of these items were consumed, it was recorded as ‘no’ iron-rich food consumption^(30)^. According to the Guide to DHS Statistics, a child is considered to have consumed iron-rich foods if they ate iron-rich foods at least once in the 24 h preceding the interview (the code of any of the following variables: v414g, v414h, v414m, v414n = 1)^(30)^, and the overall and country proportions were calculated accordingly.

The independent variables encompassed child-related factors, parental factors, household-level factors, community-level factors and country-specific factors. Child-related factors included the child’s age and sex, birth order, birth weight and breast-feeding status. The parental factors were the mother’s age, marital status, husband’s educational status, husband’s occupational status, maternal occupational status and maternal educational status.

The household-level variables were head of the household, wealth status, family size and mass media exposure. The wealth index, a cumulative measure of households’ living standards based on household assets and income, ranges from the poorest to the richest and is directly obtained from the dataset using principal component analysis. For our analysis, we recoded the variable within each country’s dataset into tertiles: ‘poor’ included the poorest and the poor quintiles, ‘middle’ continued as the middle quintile and ‘rich’ included the richest quintiles. Reproductive and other health-related variables were the timing of the first ANC visit, ANC utilisation, history of teenage pregnancy, pregnancy status at the time of the survey and number of living children (parity) of the mother.

### Data processing and statistical analysis

The datasets for each country were obtained from DHS programmes, and the data analysis was conducted using Stata 17/MP for Windows. The wealth index, ranging from poorest to richest, was directly obtained from the dataset. Due to the hierarchical structure of DHS data, the assumption of independence among observations was not met, so a hierarchical or mixed model approach was employed to address this. To evaluate the variation among the clusters (in this case, regions and clusters or EA), the intra-class correlation (ICC) coefficient and deviance values were estimated.

The outcome variable, iron-rich food consumption, was determined by the history of consuming egg, meat (beef, pork, lamb and chicken), offal meat (liver, heart and others) and fish or shellfish in the previous 24 h, as outlined in the DHS statistics guide 7.2. The overall proportion was calculated by identifying all participants with a ‘yes’ response among the total study population, and country-specific estimates were determined using the total number of participants from each country, along with their corresponding CI.

Given the substantial ICC of 29·54 % at the regional level and 41·36 % at the cluster (EA) level, we employed a two-level multilevel mixed-effects binary logistic regression to investigate factors associated with iron-rich food consumption in South and Southeast Asia countries. Before building the models, a bivariable analysis was performed to identify candidate variables for the multivariable model within each category. Four models were constructed: the null model (model I: without independent variables), model II (including only individual-level variables), model III (encompassing solely community-level variables) and model IV (incorporating both individual and community-level variables). The log-likelihood (LL) statistic, median OR (MOR) and other the indices, as shown in cluster variation analysis section of the result, were used to identify the best model.

Those variables with a *P*-value < 0·25 during the bivariable analysis were included in the multivariable binary logistic regression analysis. To evaluate multicollinearity among the independent variables, we performed a variance inflation factor analysis. The results indicated no significant multicollinearity, with all variance inflation factor values within the accepted threshold of 5 (only one subcategory of the variable had a variance inflation factor value of 5·38). To ensure robustness, we tested the model both with and without the variable, with a subcategory variance inflation factor result of 5·38 in the equation question. The results indicated that its removal did not alter either the direction or the statistical significance of the remaining independent variables. Adjusted OR (AOR) with 95 % CI were calculated for the included variables. The statistical significance of the variables and the strength of their associations were determined based on the AOR with 95 % CI.

### Parameter estimation methods

In this study, the ICC was calculated using the formula: ICC = 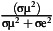 , where σμ^2^ is the variance of the group level and σe^2^ denotes the variance of the individual level^(31)^. Additionally, the proportional change in variance (PCV) was computed using the formula: PCV = 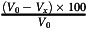 , where *V*_*0*_ is the variance of the null model and *V*_*x*_ represents the variance of each model at each level with variables^(31)^. Moreover, the MOR was calculated as follows: MOR = exp 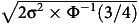 , where σ^2^ is the variance of each model and Φ^−1^ is the inverse of the standard normal cumulative distribution function^(32)^.

## Result

### Sociodemographic characteristics

This study included DHS data for 95 515 children aged 6–23 months from eleven countries in South and Southeast Asia. The mean (sd) ages of the children and mothers/caregivers were 14·35 (sd 5·11) months and 26·79 (sd 5·43) years, respectively. Of all, 63 963 (66·97 %) children were found between 12 and 23 months of age. Of the total, 85·13 % of the mothers/caregivers were between 20 and 34 years old. About one-fourth (23·97 %) of mothers in South and Southeast Asia have not attended any formal education, and over two-thirds are not engaged in paid employment, respectively. Additionally, 75·15 % of the study participants lived in rural areas, and 48·08 % of the children came from poor households. Among the study participants, 54·91 % were from higher poverty areas, 31 708 (33·20 %) lived in communities with more literate women, 26 429 (27·67 %) were from areas with higher exposure to mass media and 70 023 (73·31 %) were from communities with higher ANC utilisation.

In South and Southeast Asian countries, about one in ten women became pregnant before the age of 19 years. Of all participants, approximately 18·80 % gave birth at home to their index child. Over a quarter of respondents from South and Southeast Asia began ANC follow-ups after the first trimester. Additionally, less than one-tenth of the mothers had limited exposure to mass media. The proportions of exclusive breast-feeding and being pregnant at the time of the interview were 78·65 % and 7·38 %, respectively (Table 1).

**Table 1.** Sociodemographic and reproductive health characteristics of study participants in South and Southeast Asia, 2015–2022 (*n* 95 515)

Variable		Frequency (*n*)	Percent (%)
Parental-related study variables			
Mother’s age (in completed years)	< 20 years	4479	4·69
	20–34 years	81 316	85·13
	35–49 years	9720	10·18
Marital status	Unmarried	1363	1·43
	Married	94 152	98·57
Maternal educational status	No formal education	22 896	23·97
	Primary education	13 585	14·22
	Secondary education and above	59 034	61·81
Father’s educational status (39 419)**	No formal education	8578	21·76
	Primary education	7848	19·91
	Secondary education and above	22 993	58·33
Maternal occupation (40 137)	Not working	27 710	69·04
	Working	12 427	30·96
Father’s occupation status (39 472)**	Not working	857	2·17
	Working	38 615	97·83
Child-related study variables			
Age of the child	6–11 months	31 552	33·03
	12–23 months	63 963	66·97
Sex of the child	Female	46 116	48·28
	Male	49 399	51·72
Exclusive breast-feeding status	Yes	75 122	78·65
	No	20 393	21·35
Birth order	1	34 162	35·77
	2–4	52 160	54·61
	≥ 5	9193	9·62
Birth weight (90 713)**	Smaller than average	10 975	12·10
	Average	62 142	68·50
	Larger than average	17 596	19·40
Household-level variables			
Head of the household	Male	82 002	85·85
	Female	13 513	14·15
Family size	< 3	7885	8·26
	4–6	48 439	50·71
	≥ 7	39 191	41·03
Media exposure	Yes	7714	8·08
	No	87 801	91·92
Wealth status	Poor	45 924	48·08
	Middle	18 960	19·85
	Rich	30 631	32·07
Reproductive-related variables			
Teenage pregnancy (< 19 years)	No	85 004	89·00
	Yes	10 511	11·00
Birthplace	Health institution	77 542	81·20
	Home	17 953	18·80
Parity	< 3	79 407	83·14
	≥ 4	16 108	16·86
Antenatal care follow-up status	No antenatal care follow-up	8751	9·16
	Have antenatal care follow-up	85 657	89·68
	Do not know	1107	1·16
Time to start ANC follow-up (82 272)	< 12 weeks	59 462	72·27
	≥ 12 weeks	22 810	27·73
Plurality of birth	Single	93 955	98·37
	Multiple	1560	1·63
Preceding birth interval (61 070)*	< 23 months	14 430	23·63
	≥ 24 months	46 640	76·37
Pregnancy status at the time of the interview	No or unsure	88 465	92·62
	Yes	7050	7·38
Community or country-level			
Residence	Urban	23 735	24·85
	Rural	71 780	75·15
Country of residence	Afghanistan	8476	8·87
	Bangladesh	2482	2·6
	India	65 168	68·23
	Indonesia	5226	5·47
	Cambodia	2545	2·66
	Myanmar	1381	1·45
	Maldives	875	0·92
	Nepal	1453	1·52
	Philippines	2363	2·47
	Pakistan	3416	3·58
	Timor-Leste	2130	2·23
Country income classification	Low-income country	8476	8·87
	Upper-middle-income country	6101	6·39
	LMIC	80 938	84·74
Community-level poverty	Lower poverty	43 067	45·09
	Higher poverty	52 448	54·91
Community-level women’s literacy	Less literate	63 807	66·80
	More literate	31 708	33·20
Community-level mass media exposure	Limited exposure	69 086	72·33
	Higher exposure	26 429	27·67
Community-level ANC utilisation	Limited utilization	25 492	26·69
	Higher utilization	70 023	73·31

ANC, antenatal care; LMIC, low- and middle-income countries.

**Missing value shows the number of observations.

### The pooled proportion of iron-rich food consumption by children of 6–23 months of age

The overall proportion of iron-rich food consumption among children aged 6–23 months in South and Southeast Asia was 29·87 % (95 % CI: 29·58, 30·16). The highest intake was observed in Cambodia (79·10 %; 95 % CI: 77·46, 80·66), while the lowest was in India (20·61 %; 95 % CI: 20·29, 20·92). However, the magnitude of intake varies across countries, as shown in Table 2.

**Table 2.** Iron-rich foods consumption frequency among children of 6–23 months of age in South and Southeast Asia (*n* 95 515), 2015–2022

South and Southeast Asian countries	Survey year	Total number of children	Consumed iron-rich foods		
			Frequency (*n*)	Percentage	
				%	95 % CI
Afghanistan	2015	8476	2245	26·49	25·55, 27·44
Bangladesh	2017/18	2482	1718	69·22	67·36, 71·03
Cambodia	2021/22	2545	2013	79·10	77·46, 80·66
India	2019/21	65 168	13 428	20·61	20·29, 20·92
Indonesia	2017	5226	3646	69·77	68·50, 71·01
Maldives	2016/17	875	609	69·60	66·43, 72·63
Myanmar	2015/16	1381	782	56·63	53·96, 59·26
Nepal	2022	1453	516	35·51	33·05, 38·03
Pakistan	2017/18	3416	1247	36·50	34·89, 38·14
Philippines	2022	2363	1415	59·88	57·87, 61·86
Timor-Leste	2016	2130	914	42·91	40·79, 45·04
Total		95 515	28 533	29·87	29·58, 30·16

### Cluster variation analysis

To examine the heterogeneity in iron-rich food consumption among children aged 6–23 months across regions and EA (clusters), the analysis assessed each model using ICC and MOR. In the null model, the ICC for regions was 29·54 (95 % CI: 25·39, 34·07), and for clusters, it was 41·36 (95 % CI: 37·69, 45·13), indicating substantial heterogeneity in iron-rich food consumption across the geographic levels in South and Southeast Asia. The presence of heterogeneity across regions and EA was further confirmed by the MOR values: 3·29 (95 % CI: 2·40, 4·52) at the regional level and 2·16 (95 % CI: 2·04, 2·29) at the EA level. This means that, if two children were randomly selected from different regions, the child in the region with higher iron-rich food consumption would be 3·29 times more likely to consume such foods than a child from a region with lower consumption. Similarly, the odds of consuming iron-rich foods among children in different EA can lead to 2·16 times differences in such food consumption among children, indicating notable variation at the local level.

Table 3 presents the model comparison parameters, including LL, MOR and deviance, which were used to assess the models that best fit the data. Model IV demonstrated the best fit, evidenced by the lowest negative LL value (–44624·24) and the lowest Akaike information criterion score (89320·49).

**Table 3.** Variation in the fitness of each model for iron-rich food consumption among children aged 6–23 months in South and Southeast Asian countries, 2015–2020

Parameters		Model I		Model II		Model III		Model IV	
ICC (%, 95% CI)	Region level	29·54	25·39, 34·07						
	EA level	41·36	37·69, 45·13						
Variance (95 % CI)	Region level	1·56	1·27, 1·91	1·47	1·45, 2·18	0·36	0·29, 0·45	0·40	0·32, 0·49
	EA level	0·65	0·60, 0·71	0·69	0·63, 0·75	0·65	0·60, 0·71	0·69	0·63, 0·75
PCV (%)	Region level	Reference		14·09		76·76		74·27	
	EA level	Reference		05·15		0·56		4·83	
MOR (95 % CI)	Region level	3·29	2·40, 4·52	3·57	2·48, 5·13	1·78	1·64, 1·93	1·83	1·67, 1·99
	EA level	2·16	2·04, 2·29	2·21	2·08, 2·35	2·16	2·04, 2·29	2·21	2·08, 2·35
LL (AIC)		−46980·48	93 966·96	−44776·15	89 594·3	−46828·61	93 693·21	−44624·24	89 320·49
Deviance (LLR test *v.* logistic model)		22525·82		21932·05		6219·59		6448·97	

ICC, inter-class correlation; EA, enumeration areas; PCV, proportional change in variance; MOR, median OR; LL, log-likelihood; LLR, log-likelihood ratio; AIC, Akaike information criterion; model I, null model; model II, adjusted for individual-level variables; model III, adjusted for household-level variables, model IV, full/final model (adjusted for individual- and household-level variable).

### Factors associated with iron-rich food consumption

Before conducting multivariable analysis, a bivariable multilevel mixed-effects logistic regression was used to identify factors associated with the consumption of iron-rich foods among children aged 6–23 months in South and Southeast Asian countries. An independent analysis was conducted for each study variable in relation to iron-rich food consumption.

In the bivariable analysis, individual-level variables including child’s age, sex, plurality, exclusive breast-feeding status, respondent’s age, history of teenage pregnancy, wealth index, place of delivery, current pregnancy status (at the time of interview), birth order, sex of the household head, mass media exposure and ANC utilisation were identified as candidates (*P*-value ≤ 0·25) for the multivariable analysis. In addition, community-level variables such as country, place of residence, community-level poverty, women’s literacy, mass media exposure and ANC utilisation were also selected for inclusion at the multivariable analysis stage (see online supplementary material, Supplemental Table 1).

All these variables were incorporated into different multivariable, multilevel, mixed-effects binary logistic regression models (two-level), ranging from model II to model IV, as shown in Table 4. Finally, the final multivariable analyses model (model IV) revealed that the age of the child, exclusive breast-feeding status, history of teenage pregnancy, place of delivery, pregnancy status (at the time of the interview), birth order, sex of the household head and mass media exposure were associated with iron-rich food consumption among children aged 6–23 months in South and Southeast Asian countries. Furthermore, country of origin, place of residence, community-level women’s literacy, mass media exposure and ANC utilisation were statistically significant predictors of iron-rich food consumption in the final model.

**Table 4.** Multilevel mixed-effects binary logistic regression analysis examining the factors associated with iron-rich food consumption among children aged 6–23 months in South and Southeast Asia from 2015 to 2022

Variables		Model I (null model)	Model II		Model III		Model IV	
			AOR	95 % CI	AOR	95 % CI	AOR	95 % CI
Age of the child	6–11 months		1				1	
	12–23 months		3·59	3·44, 3·75			3·59	3·45, 3·76*
Sex of the child	Female		1				1	
	Male		0·97	0·94, 1·01			0·97	0·94, 1·01
Plurality	Single		1				1	
	Multiple		0·91	0·79, 1·06			0·91	0·79, 1·06
Exclusive breast-feeding status	No		1				1	
	Yes		1·17	1·11, 1·22			1·17	1·12, 1·23*
Respondents age	< 20 years		1				1	
	20–34 years		1·10	0·99, 1·23			1·10	0·99, 1·23
	35–49 years		1·08	0·96, 1·23			1·07	0·95, 1·22
Teenage pregnancy	Not teen		1				1	
	Teen		1·10	1·02, 1·19			1·10	1·02, 1·19*
Place of delivery	Home		1				1	
	Health institution		1·11	1·05, 1·17			1·10	1·04, 1·16*
Currently pregnant (at the time of interview)	No		1				1	
	Yes		1·59	1·49, 1·71			1·60	1·50, 1·72*
Birth order	1		1				1	
	2–4		1·26	1·21, 1·32			1·26	1·20, 1·32*
	≥ 5		1·29	1·17, 1·42			1·29	1·18, 1·43*
Parity	< 4		1				1	
	≥ 4		0·94	0·88, 1·01			0·94	0·88, 1·01
Sex of the household head	Male		1				1	
	Female		1·07	1·02, 1·13			1·06	1·01, 1·12*
Mass media exposure	No		1				1	
	Yes		1·29	1·21, 1·38			1·27	1·19, 1·36*
ANC utilisation	No		1				1	
	Yes		1·06	0·99, 1·14			1·05	0·98, 1·13
	Do not know		1·04	0·88, 1·24			1·04	0·87, 1·24
Wealth status	Poor		1				1	
	Middle		0·99	0·94, 1·04			0·97	0·93, 1·03
	Rich		1·07	1·01, 1·12			1·03	0·98, 1·09
Country	Afghanistan				1		1	
	Bangladesh				9·34	5·52, 15·79	11·82	6·79, 20·55*
	India				1·02	0·69, 1·51	1·09	0·72, 1·65
	Indonesia				5·64	3·47, 9·17	6·78	4·07, 11·31*
	Cambodia				15·55	9·99, 24·21	20·72	13·00, 33·02*
	Myanmar				3·12	1·88, 5·20	3·72	2·18, 6·36*
	Maldives				5·30	2·77, 10·14	5·83	2·95, 11·53*
	Nepal				1·45	0·79, 2·66	1·52	0·80, 2·89
	Philippines				3·37	1·97, 5·77	4·05	2·30, 7·12*
	Pakistan				1·88	1·14, 3·12	2·06	1·21, 3·49*
	Timor-Leste				1·96	1·24, 3·11	2·25	1·39, 3·64*
Residence	Urban				1		1	
	Rural				0·88	0·84, 0·92	0·88	0·84, 0·94*
Community-level poverty	Lower poverty				1		1	
	Higher poverty				1·18	0·97, 1·44	1·18	0·97, 1·45
Community-level Women’s literacy	Less literate				1		1	
	More literate				0·38	0·23, 0·64	0·36	0·21, 0·62*
Community-level mass media exposure	Limited exposure				1		1	
	Higher exposure				1·66	1·22, 2·24	1·67	1·21, 2·29*
Community-level ANC utilisation	Limited utilisation				1		1	
	Higher utilisation				1·02	0·77, 1·34	1·01	0·75, 1·34

AOR, adjusted OR; ANC, antenatal care; NB, model I is the null model.

*P-value < 0·05.

Children aged 12–23 months were 3·59 times (95 % CI: 3·45, 3·76) more likely to consume iron-rich foods than children younger than 12 months. Also, a child with a history of exclusive breast-feeding had 1·17 times higher odds (95 % CI: 1·12, 1·23) of having iron-rich foods than those without exclusive breast-feeding.

Children born to teenage mothers were 1·09 (95 % CI: 1·02, 1·17) times more likely to consume iron-rich foods compared to their counterparts. Additionally, children born in healthcare institutions were 10 % (AOR = 1·10, 95 % CI: 1·02, 1·19) more likely to consume iron-rich foods compared to those born at home. Moreover, children whose mothers were pregnant at the time of the interview were 1·60 times (95 % CI: 1·50, 1·72) more likely to consume iron-rich foods compared to children whose mothers were not pregnant during the interview.

Similarly, children born with a birth order of 2–4 had 26 % higher odds (AOR = 1·26, 95 % CI: 1·20, 1·32) of consuming iron-rich foods, while those with a birth order of 5 or more had 29 % higher odds (AOR = 1·29, 95 % CI: 1·18, 1·43) compared to firstborn children. Additionally, children from female-headed households exhibited a 6 % increase in the odds (AOR = 1·06, 95 % CI: 1·01, 1·12) of iron-rich food consumption as compared to male-headed households. Likewise, children from households exposed to mass media were 1·27 times more likely (95 % CI: 1·19, 1·36) to consume iron-rich foods compared to those from households without such exposure.

Furthermore, compared to children in Afghanistan, children in Cambodia had the highest likelihood of consuming iron-rich foods (AOR = 20·72; 95 % CI: 13·00, 33·02), followed by Bangladesh (AOR = 11·82; 95 % CI: 6·79, 20·55), Indonesia (AOR = 6·78; 95 % CI: 4·07, 11·31), Maldives (AOR = 5·83; 95 % CI: 2·95, 11·53) and the Philippines (AOR = 4·05; 95 % CI: 2·30, 7·12). Children in Myanmar (AOR = 3·72; 95 % CI: 2·18, 6·36), Timor-Leste (AOR = 2·25; 95 % CI: 1·39, 3·64) and Pakistan (AOR = 2·06; 95 % CI: 1·21, 3·49) also had significantly higher odds of iron-rich food consumption compared to Afghanistan.

Children living in rural areas were 12 % less likely (AOR = 0·88, 95 % CI: 0·84, 0·84) to consume iron-rich foods compared to their urban counterparts. Additionally, children residing in communities with higher levels of women’s literacy had significantly lower odds of consuming iron-rich foods compared to those in less literate communities (AOR = 0·36; 95 % CI: 0·21, 0·62). In contrast, children from communities with greater mass media exposure were significantly more likely to consume iron-rich foods than those in communities with limited media exposure (AOR = 1·67; 95 % CI: 1·21, 2·29).

## Discussion

This study examines the prevalence of iron-rich food consumption and its associated factors among children aged 6–23 months, utilising recent DHS data from South and Southeast Asian countries. It reveals two key findings: first, the consumption of iron-rich foods was generally low. Second, consumption patterns were influenced by individual (maternal and child sociodemographic, reproductive) and community-level factors.

The overall magnitude of iron-rich food consumption among children aged 6–23 months in South and Southeast Asia was 29·87 % (95 % CI: 29·58, 30·16), with a range of variability across countries in the region, from 79·10 % in Cambodia to 20·61 % in India. The proportion of iron-rich food consumption is relatively low when compared to a similar study in SSA countries (42·1 %)^(25)^. Also, this magnitude is lower than the result of studies conducted in other low- and middle-income countries, like Mexico (63·1 %)^(33)^, East Asia and the Pacific (62·5 %)^(34)^ and China (51 %)^(35)^. The possible reason for this variation in iron-rich food consumption across the region and countries may be attributed to household food insecurity and the poor economic status of South and Southeast Asian countries.

This economic limitation could result in higher prices for iron-rich foods than non-iron-rich options, such as ultra-processed foods^(36)^. Additionally, cultural taboos and religious factors likely influence the consumption of meat and organ meat^(37)^. The present study reveals that children’s consumption of iron-rich foods varies based on their country of residence, as highlighted by various studies in South and Southeast Asia^(3,38)^. Factors such as cultural dietary practices, the availability of food sources and socio-economic conditions within each country likely contribute to the observed differences in iron-rich food consumption among children. The low consumption of iron-rich foods among children aged 6–23 months in South and Southeast Asia mirrors similar dietary patterns at the household level. Therefore, to reduce the prevalence and consequences of iron deficiency anaemia in the region, nutrition programmes should prioritise increasing the intake of iron-rich foods, including both animal-based and alternative sources of iron.

In this study, children aged 12–23 months consumed more iron-rich foods than younger children. This finding aligns with studies from Mexico^(39)^ and SSA^(25)^. The reason may be mothers’ knowledge and attitudes about feeding practices, as well as cultural perceptions or taboos regarding the consumption of animal food by children under 1 year. Additionally, children are more likely to eat the same foods prepared for the rest of the family or adults after their first year.

Additionally, children who were exclusively breastfed were more likely to consume iron-rich foods than those who were not, consistent with findings from a study in Sierra Leone^(40)^. In South Asia, however, only 60 % of mothers practice exclusive breast-feeding^(41)^, with many introducing other foods and liquids early^(39,41)^. This early introduction of foods and liquids can expose children to pathogens and digestive issues, potentially leading to illness. As a result, some mothers may avoid giving iron-rich foods to their children, either due to early negative associations or a lack of understanding about their benefits, as documented in studies examining iron intake, breast-feeding and feeding practices in various settings^(42)^.

This study found that maternal and reproductive health characteristics were associated with the consumption of iron-rich foods. Notably, children of mothers with a history of teenage pregnancy had higher consumption of iron-rich foods compared to their counterparts, consistent with findings from a study conducted in Brazil^(43)^. One possible explanation is that mothers who experienced a teenage pregnancy might have a heightened awareness of nutritional deficiencies, potentially due to targeted education or interventions received during healthcare visits. This knowledge could lead mothers to prioritise the provision of iron-rich foods to their children^(33,40)^. Additionally, these mothers might be more engaged with ANC healthcare services, resulting in better adherence to dietary recommendations. Cultural or familial practices in communities with higher rates of teenage pregnancy might also emphasise traditional diets rich in iron, influencing their children’s eating habits^(44)^.

Additionally, children born in healthcare institutions, children whose mothers were pregnant at the time of the interview and children born with a birth order of 2 or more had higher consumption of iron-rich foods compared to their counterparts, which is in line with the findings of earlier studies conducted in Africa^(21,25)^ and Pakistan^(42)^. These factors may be linked to improved maternal knowledge and attitudes through frequent visits and nutrition education by healthcare providers. This nutrition education helps mothers understand the causes of iron deficiency anaemia and how to prevent it by consuming iron-rich foods for both mothers and their children^(45)^.

Children from female-headed households were found to consume more iron-rich foods. While such households often face economic disadvantages^(46)^, their impact on child nutrition appears to vary. Some studies have found better nutritional outcomes in female-headed households, including higher consumption of iron-rich foods and more diverse diets, particularly among low-income groups^(47)^. However, other studies have reported higher rates of child malnutrition in these households^(46)^. Factors such as women’s control over resources, decision-making roles and food distribution within the household have a significant influence on these outcomes. The mixed findings suggest that the relationship between household headship and child nutrition is complex and context-dependent.

On the other hand, children from households exposed to mass media had significantly higher consumption of iron-rich foods. This finding is similar to previous studies using DHS in Ethiopia, East Africa, Rwanda and SSA^(21,23,25)^. Moreover, a systematic review revealed that mass media exposure increases the complementary feeding practices of mothers when it is combined with nutrition education^(48)^. The role of mass media in providing mothers or caregivers with health messages on optimal feeding practices for children has been shown to significantly enhance mothers’/caregivers’ knowledge and behaviours regarding IYCF^(49)^. This suggests that improving women’s access to media could contribute to increased consumption of iron-rich foods among children aged 6–23 months in these countries and regions.

Children from households residing in urban areas had a higher consumption of iron-rich foods than those living in rural areas. The findings on the disparities in dietary habits and micronutrient intake between rural and urban regions align with those from other studies conducted in Indonesia^(50)^, India^(51)^ and Pakistan^(52)^. One possible explanation is that children from urban households may have better access to a wider variety of foods through supermarkets, grocery stores and restaurants. Additionally, urban lifestyles often promote greater nutrition and health awareness through education and awareness campaigns, positively influencing dietary choices.

This study reveals significant cross-country variation in the consumption of iron-rich foods, consistent with earlier research that reported similar disparities across national contexts^(16)^. These differences are influenced by various factors, including agricultural productivity, which plays a critical role in determining access to nutrient-rich foods, particularly in low- and middle-income countries where food systems are predominantly localised. A country’s economic status has a significant impact on household food security and the ability to purchase a diverse range of iron-rich foods. Additionally, government policies, strategies and public health interventions play a crucial role in shaping dietary patterns and supporting IYCF practices. Countries that implement targeted nutrition programmes, food fortification initiatives and health promotion campaigns are more likely to follow dietary guidelines more closely.

Children residing in communities with higher levels of women’s literacy were found to have significantly lower odds of consuming iron-rich foods, which is an observation that contradicts findings from other studies^(17,22)^. Several factors may explain why children residing in communities with higher levels of female literacy are less likely to consume iron-rich foods. Although educated women are generally expected to have better knowledge, attitudes and practices related to IYCF, this does not always translate into improved nutritional behaviour at the community level. Residing in communities with a high level of women’s literacy may not be sufficient to overcome entrenched cultural or religious dietary restrictions, such as the avoidance of meat as well as persistent economic barriers, high food prices and intra-household decision-making dynamics. All these factors can limit access to and consumption of iron-rich foods, regardless of the women’s literacy level in the community.

In contrast, individuals from communities with greater mass media exposure were significantly more likely to consume iron-rich foods than those with limited exposure, which is consistent with a previous similar study in SSA^(25)^ and other research findings^(48,53)^. Mass media likely plays a pivotal role in shaping caregivers/mothers’ knowledge, attitudes and practices related to IYCF by providing persuasive health information.

As a result, implementing or enhancing nutrition-specific interventions, such as targeted counselling, iron supplementation and social safety net measures, for both short-term and long-term strategies, including food system diversification and maternal education, is crucial to sustainably increase the intake of iron-rich foods among young children in the region.

### Study strengths and limitations

The major strengths of this study include the use of nationally representative data and a large sample size from South and Southeast Asian countries. However, several limitations should be acknowledged. The analysis relies on cross-sectional survey datasets, which limit the ability to establish definitive causal relationships between various factors and iron-rich food consumption among children. Additionally, the assessment of iron-rich food intake was based on a binary (Yes/No) recall of consumption within the previous 24 h, which may not accurately capture habitual dietary patterns. This may not represent typical intake, especially for micronutrients such as iron and vitamin A, given that some food items, which are major sources of these micronutrients (such as liver), are consumed very irregularly.

Our study may also have underestimated the consumption of iron-rich foods by excluding plant-based sources of iron. Furthermore, iron-fortified foods and dietary factors that enhance or inhibit iron absorption were not assessed. We did not account for the recommended daily allowance or the iron content of specific foods in our analysis, as these variables were either missing or not available in the datasets.

Future research should focus on employing more comprehensive and specific measures of iron-rich diet consumption, considering plant-based iron sources, dietary factors that inhibit or enhance iron absorption and additional iron sources such as supplements and fortified foods. Dietary intake assessment tools, such as semi-quantitative FFQ and/or multiple 24-h dietary recalls, can provide more accurate quantitative estimates of iron intake, allowing for comparison with the RDA for specific age groups and providing a clearer understanding of iron consumption patterns. Acknowledging the differences in dietary habits, cultural practices and food preferences among countries will also be beneficial, as it can provide context-specific information for policy formulation.

### Conclusion

This study reveals that only about one-third of children aged 6–23 months in the South and South Asian regions consume iron-rich foods. However, there is wide variation across countries, with the proportion ranging from 79·1 % in Cambodia to just 20·6 % in India. Improving the consumption of iron-rich foods among children aged 6–23 years is crucial in this region, as reviewing and strengthening strategies to effectively address inadequate iron intake among young children. Alongside short-term interventions such as iron supplementation, increasing awareness about nutrition and health through various media platforms can improve IYCF practices and, consequently, promote the regular consumption of an iron-rich diet.

## Supporting information

Engidaw et al. supplementary material 1 Engidaw et al. supplementary material

Engidaw et al. supplementary material 2 Engidaw et al. supplementary material

## Data Availability

The datasets are accessible to the public and can be downloaded from the DHS programme with permission. Anyone can obtain each country’s dataset through https://dhsprogram.com/data/available-datasets.cfm.https://dhsprogram.com/data/available-datasets.cfm. The compiled dataset used and/or analysed during the current study is available from the corresponding author upon reasonable request.

## Supplementary material

For supplementary material accompanying this paper visit https://doi.org/10.1017/S1368980025101626.
