# Supplementary material for: Iron-rich foods consumption and its associated factors among children aged 6–23 months in South and Southeast Asia: a multilevel analysis of demographic and health surveys
Source: Public Health Nutr. 2025 Dec 26;29(1):e20. doi: 10.1017/S1368980025101626 (PMC12895479; doi:10.1017/S1368980025101626)
Supplement: Engidaw et al. supplementary material 1 — Engidaw et al. supplementary material [file S1368980025101626sup001.docx]

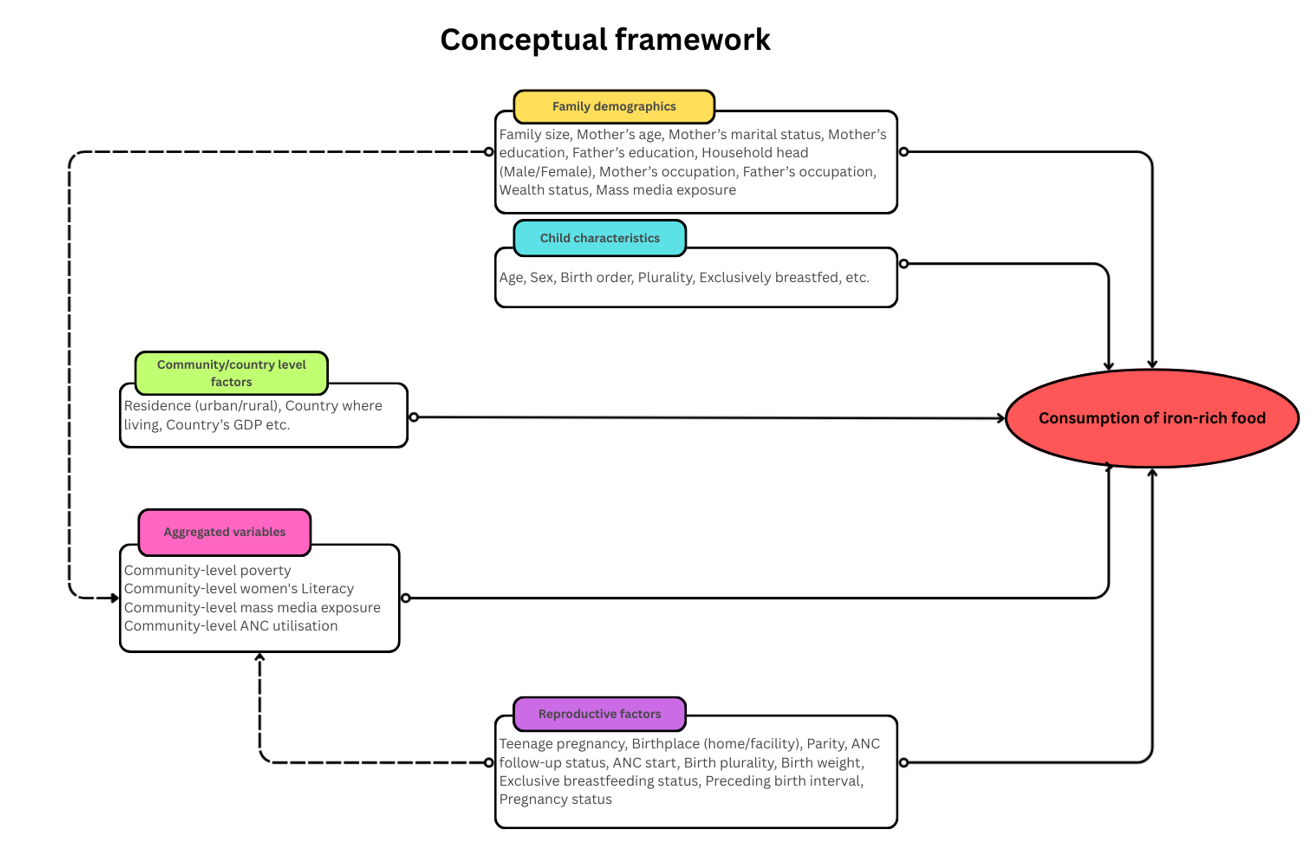


**Supplementary Figure 1:** Conceptual framework of factors influencing iron-rich food consumption, modified from Akalu et al (25).
