# Supplementary material for: Iron-rich foods consumption and its associated factors among children aged 6–23 months in South and Southeast Asia: a multilevel analysis of demographic and health surveys
Source: Public Health Nutr. 2025 Dec 26;29(1):e20. doi: 10.1017/S1368980025101626 (PMC12895479; doi:10.1017/S1368980025101626)
Supplement: Engidaw et al. supplementary material 2 — Engidaw et al. supplementary material [file S1368980025101626sup002.docx]

**Supplementary Table 1**: Bivariable multilevel mixed-effects logistic regression analysis of factors influencing iron-rich food consumption among 6–23 months children in South and Southeast Asia (2015–2022).

| **Variables** | | **COR 95% CI** | **P – value** |
| --- | --- | --- | --- |
| Age of the child | 06 – 11 months | 1 | <0.001 |
|  | 12 – 23 months | 3.60(3.45, 3.76) |  |
| Sex of the child | Female | 1 | 0.092 |
|  | Male | 0.97(0.94, 1.01) |  |
| Plurality | Single | 1 | 0.096 |
|  | Multiple | 0.88(0.77, 1.02) |  |
| Exclusive Breastfeeding status | Yes | 1 | <0.001 |
|  | No | 0.83(0.80, 0.87) |  |
| Respondents age | <20 years | 1 | <0.001 |
|  | 20 -34 years | 1.29(1.19, 1.40) |  |
|  | 35-49 Years | 1.34(1.22, 1.48) |  |
| Place of delivery | Home | 1 | 0.004 |
|  | Health Institution | 1.07(1.02, 1.34) |  |
| Teenage pregnancy | Not teen | 1 | 0.191 |
|  | Teen | 0.96(0.91, 1.02) |  |
| Currently pregnant (at the time of interview) | No | 1 | <0.001 |
|  | Yes | 1.88(1.75, 2.00) |  |
| Birth order | 1 | 1 | <0.001 |
|  | 2 – 4 | 1.19 (1.15, 1.24) |  |
|  | ≥ 5 | 1.12(1.05, 1.20) |  |
| Parity | < 4 | 1 |  |
|  | ≥ 4 | 1.04(0.99, 1.09) |  |
| Sex of the household head | Male | 1 | 0.041 |
|  | Female | 1.05(1.00, 1.11) |  |
| Mass media exposure | No | 1 | <0.001 |
|  | Yes | 1.26(1.18, 1.34) |  |
| ANC utilisation | No | 1 | 0.122  0.744 |
|  | Yes | 1.05(0.99, 1.13) |  |
|  | Do not know | 1.02(0.87, 1.22) |  |
| Wealth Index | Poor | 1 | 0.552  0.021 |
|  | Middle | 0.98(0.94, 1.03) |  |
|  | Rich | 1.06(1.01, 1.11) |  |
| Residence | Urban | 1 | < 0.001 |
|  | Rural | 0.88(0.83, 0.92) |  |
| Country | Afghanistan | 1 | <0.001 except India = 0.945, and Pakistan = 0.011 |
|  | Bangladesh | 9.41 (5.58, 15.87) |  |
|  | India | 1.01 (0.73, 1.39) |  |
|  | Indonesia | 9.86 (7.60, 13.76) |  |
|  | Cambodia | 16.83 (11.63, 24.37) |  |
|  | Myanmar | 5.11 (3.32, 7.86) |  |
|  | Maldives | 9.27 (5.06, 16.98) |  |
|  | Nepal | 2.07 (1.19, 3.62) |  |
|  | Philippines | 5.87(3.92, 8.80) |  |
|  | Pakistan | 1.96 (1.16, 3.30) |  |
|  | Timor-Leste | 2.63 (1.69, 4.09) |  |
| Community-level poverty | Lower poverty | 1 | 0.055 |
|  | Higher poverty | 1.41(0.99, 1.99) |  |
| Community-level Women's Literacy | Less literate | 1 | <0.001 |
|  | More literate | 0.12(0.05, 0.28) |  |
| Community-level Mass Media Exposure | Limited exposure | 1 | <0.001 |
|  | Higher exposure | 2.43(1.75, 3.74) |  |
| Community-level ANC Utilisation | Limited utilization | 1 | <0.001 |
|  | Higher utilization | 2.66(1.88, 3.76) |  |
| **COR**: Crude Odds Ratio; **CI**: Confidence Interval; | | | |
